# Retrospective Analysis of Triage and Hospitalisation Records for Bushfire-Affected Koalas (*Phascolarctos cinereus*) and Other Wildlife Species from Victoria, Australia, 2019–2020

**DOI:** 10.3390/ani16060944

**Published:** 2026-03-17

**Authors:** Caitlin N. Pfeiffer, Bonnie McMeekin, Lee F. Skerratt, Richard J. Ploeg

**Affiliations:** 1 Melbourne Veterinary School, University of Melbourne, Werribee, VIC 3030, Australia; l.skerratt@unimelb.edu.au (L.F.S.); richard.ploeg@csiro.au (R.J.P.); 2 Zoos Victoria, Parkville, VIC 3052, Australia; bmcmeekin@zoo.org.au

**Keywords:** bushfire, wildfire, koala, *Phascolarctos cinereus*, burn, mortality, hospitalisation, survival, prognosis

## Abstract

Following bushfires (also known as wildfires), impacted free-living wildlife with welfare or conservation concerns are captured and presented for veterinary assessment where possible. This study represents an in-depth retrospective analysis of the veterinary records of 259 animals in Victoria, Australia, impacted by bushfire in 2019–2020. In total, 35 different species were assessed, including 196 koalas. Multivariable analyses of 126 koalas with complete medical records identified several clinical prognostic factors affecting 6-month survival outcomes. Increased odds of negative outcomes (death or euthanasia) were associated with increasing age (tooth wear class; odds ratio 2.70 for one unit increase), lower body condition score (one-unit decrease OR 7.27), and the earlier animals were presented after the fire event (OR 0.94 for each passing day). In 83 koalas with burn injuries, negative outcomes were also associated with burns more severe than minor (85% survival for minor burns only, compared to 31% survival with moderate or severe burns), and burns to more than 10 digits (12% survival). In burnt koalas, the combination of burn severity and digital involvement appear to be important prognostic factors for long-term outcomes. These findings can support veterinarians to more accurately evaluate prognosis for bushfire-affected koalas during initial assessment and will facilitate the strategic allocation of limited treatment and rehabilitation resources to the animals most likely to recover. The scope of this study was limited to the consideration of health outcomes, with the recognition of health as just one of many factors that must inform decisions about rehabilitating injured wildlife.

## Simple Summary

Following bushfires (also known as wildfires), impacted free-living wildlife may be captured and presented for veterinary assessment. This study was an in-depth investigation of the records of veterinary assessment and treatment of 259 animals in Victoria, Australia, impacted by bushfire in late 2019 and early 2020. The animals assessed included 35 different species, most commonly koalas. Further evaluation of the 6-month post-rescue outcomes for koalas presented for assessment was undertaken to understand the likelihood of survival for koalas with health impacts of fire exposure including injuries. The koalas least likely to survive were older koalas, koalas in poor body condition, and those rescued and evaluated in the first days after the fire. Two thirds of the koalas had burn injuries, and in addition to age and body condition, those with more severe burns and burns to many toes were least likely to survive. These findings can support veterinarians in understanding the likely medical outcomes for bushfire-affected koalas in the future and thus also assist in the appropriate allocation of treatment and rehabilitation resources to those animals most likely to recover.

## 1. Introduction

The Australian bushfires (also known as wildfires) of late 2019 and early 2020 drew worldwide attention, not only for the associated destruction of many towns and communities which stood in their path, but also the devastating impact on wildlife populations, including the iconic Australian koala (*Phascolarctos cinereus*) [1]. At the time of these fires, the south-eastern state of Victoria was in its third consecutive year of rainfall deficit [2]. During the 2019–2020 fire season, a total of 1.58 million hectares of Victorian land was burnt, the largest area in Victoria to be bushfire-impacted in a single season since 1939 [3]. The burnt area was 91% native forest, comprising 19% of the total native forest in Victoria [4], and Geary et al. [5] identified 346 species in Victoria that had >40% of their modelled habitat affected by the fire, including 45 threatened species. In the East Gippsland region, a series of fires were ignited between 18 and 21 November 2019, growing to a combined area of 2350 ha by 25 November. Further fires in East Gippsland were ignited on 20 December, and over subsequent days several smaller fires combined to create large fires in the area. On 30 December, multiple fires started from dry lightning, and three fires in East Gippsland remained active with a combined area of over 130,000 ha, with a total burnt area in East Gippsland of 230,000 ha by the end of December 2019. A State of Disaster was declared by the Victorian Government on 2 January 2020. Widespread rain eased fire conditions on 20 and 22 January 2020, although uncontrolled bushfires in some areas of East Gippsland continued to burn until the final major fire was declared contained on 27 February [6].

Many organisations and individuals were mobilised to provide care to the thousands of bushfire-affected animals. The response to the injured wildlife in Victoria, described in detail in Parrott et al. [1], was led by the Victorian State Government Department of Environment, Land, Water and Planning (DELWP) (now the Department of Energy, Environment and Climate Action—DEECA) which responded in partnership with Zoos Victoria (ZV). To facilitate veterinary assessment and initial care of injured wildlife, DELWP/ZV utilising a support vehicle and equipment via the Royal Society for the Prevention of Cruelty to Animals set up three field stations in early January 2020 to allow for the triaging of affected animals. These were located at Mallacoota, Bairnsdale and Corryong. The Bairnsdale field station was re-located to Gelantipy for a short period from the 6th to 8th of February. Each field station had a DELWP/ZV veterinary team consisting of one veterinarian, one nurse and, in some instances, a zookeeper. Almost 3000 animals in bushfire-affected areas were initially assessed in situ, some required euthanasia on humane grounds, some were left in place and others were translocated to a nearby unburnt habitat [1]. Animals that were not suitable to be left in place or translocated, but not sufficiently injured to require immediate euthanasia, were presented to the field stations. After a more thorough assessment by the veterinary teams, a subset of animals were subsequently transferred to ZV’s wildlife hospitals for more intensive care and management.

In the process of treatment at field stations, records of clinical assessments were created for each animal presented. Animals that were subsequently transferred for intensive management in ZV wildlife hospitals also had more detailed clinical and treatment data recorded; in most cases, these records detailed their management longitudinally to the point of release to the wild. These detailed records provided an opportunity to evaluate a host of clinical parameters and therapeutic interventions and assess their association with outcomes for bushfire-injured wildlife.

Clinical parameters or therapeutic interventions associated with long-term outcomes may serve as prognostic indicators, which can inform clinical assessments at field stations or during the early stages of hospitalisation. Better understanding of factors associated with survival can facilitate strategic selection of cases for intensive treatment and underpin clinical judgements about long-term prognosis. Therefore, this retrospective study of clinical records, investigating the 259 animals presented to field stations reported by Parrott et al. [1], aimed to identify clinical or therapeutic factors associated with survival and release of bushfire-injured wildlife. Given 196 presented animals were koalas, additional analyses focussed on factors specifically affecting survival of rescued koalas including those with burn injuries. While the results of this retrospective study cannot identify prognostic indicators that predict the outcome for every bushfire-affected koala, it is hoped that this investigation will provide evidence to inform objective decision making by veterinarians, to make efficient use of rehabilitation resources, and minimise suffering for bushfire-affected wildlife.

## 2. Materials and Methods

### 2.1. Field Station Records

**Figure 1:**
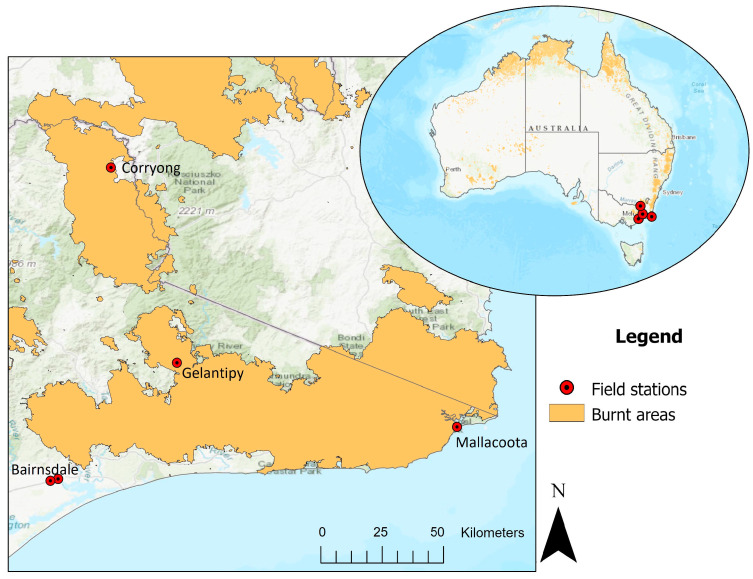
Map showing the extent of burnt forest in eastern Victoria, Australia during the 2019–2020 fire season, with point locations of the field stations where wildlife was assessed and treated by veterinarians. Inset map shows the national bushfire extent in Australia, 2019–2020.

At the three field stations receiving wildlife (Bairnsdale including Gelantipy, Mallacoota, and Corryong, as shown in Figure 1), veterinarians examined animals burned or otherwise impacted by fire and provided field medical treatment (‘first aid’). Field stations were staffed by experienced wildlife veterinarians from ZV and other institutions with additional support by experienced DELWP Forest and Wildlife officers. The field stations were in operation for a total period of 52 days (1 January 2020 to 21 February 2020). Following initial health assessment, animals either received appropriate initial medical treatment or were euthanised. Euthanasia was conducted on welfare grounds, where prognosis was assessed as poor either due to fire-related injuries or other comorbidities. Animals assessed as having a reasonable prognosis for recovery following initial medical treatment received one of three outcomes: (1) released, where no ongoing care or treatment was deemed necessary; (2) dispatched to suitable authorised wildlife shelters for short-term care and rehabilitation; or (3) transported to ZV hospital facilities for further necessary medical management.

At initial presentation to the field station, all wildlife were examined and for each individual animal, relevant clinical data was entered in DELWP animal triage record books. The information registered included the field station, the location the animal was found, species, date and time of presentation, any specific identifying features, sex, weight, body condition score (BCS) [7] and age. Age was estimated for koalas based on the correlation with tooth wear class (TWC) [8,9], while for all other species age was recorded as juvenile or adult based on physical appearance.

External lesions or burns were also recorded at initial examination for all species, including the body region affected (head, thorax, abdomen, limbs) and the relative proportion of each region affected. More in-depth information on burn lesion distribution for specific regions were also noted: for the head, involvement of the ears, eyes, nostrils, or mouth; for the torso, assessment of the pouch and genitals; and for each injured limb (i.e., left fore, right fore, left hind, right hind) details of involvement of the palm, dorsal surface of the paw, and the number of digits affected. For each burn lesion, severity was subjectively graded by the clinician using a variety of terminology (Table 1), as is typical of bushfire responses with multiple attending clinicians involved [10].

Other clinical examination findings recorded included rectal temperature, heart rate, respiration rate and pattern, hydration status (based on skin glide over scapula and skin tenting over top of head [7]) and mucous membrane colour. In addition to clinical observations, initial therapeutic interventions were recorded in detail, including sedatives, antibiotics or analgesics administered, and both type and route of administration of fluid therapy. The outcome following triage was recorded for each animal (died, euthanised, released, or transferred including destination of transfer).

**Table 1:** Burn scores derived from triage and clinical records for koalas with burn injuries presented to field stations in Victoria, Australia, during 2020.

Burn Score Descriptor	Burn Score	Burn Score Definition	Example Terms Used by Veterinarians in Triage and Clinical Records	Expected Medical Description
No burns	Grade 0	No burns present	OK, nil, NAD, no burns, normal, blank, no lesions, perfect feet.	Clinically normal healthy skin.
Minor	Grade 1	Only minor burns present	minor, mild, superficial, pink, singed, slight, depigmentation, healing, grade 1.	Superficial burn: Involving the outermost skin. Could also be described by terminology ‘first-degree burn’.
Minor to moderate	Grade 1.5	Majority of burns minor; at least one burn described consistent with moderate	N/A	N/A
Moderate	Grade 2	Majority of burns moderate; no burns described consistent with severe	partial thickness, second degree burn, moderate, grade 2.	Partial thickness burn: Involving deeper levels of the skin. Could also be described by terminology ‘second-degree burn’.
Moderate to severe	Grade 2.5	Majority of burns moderate; at least one burn described consistent with severe	N/A	N/A
Severe	Grade 3	Majority of burns severe	full thickness, severe, sloughing skin, bad, significant, detaching toe/nail, bone exposed, grade 3.	Full thickness burn: Destroys the full depth of skin, including tissues below. Could also be described by terminology ‘third-degree burn’.

### 2.2. Hospital Medical Records

After initial veterinary assessment, 33 animals were transferred to ZV wildlife hospitals for further treatment, including 29 koalas. For animals transferred to ZV, detailed medical records were kept via the institution’s information management system ZIMS (Zoological Information Management System, https://species360.org/zims/, accessed on 6 November 2025). Clinical pathology testing was undertaken for each case as required based on their clinical management, with urinalysis (Multistix^®^ 10 SG, Siemens, PA, USA) performed in-house at ZV, blood gas analysis performed externally at Gribbles Veterinary Pathology, Clayton, Victoria, and haematology and biochemistry performed either at Gribbles Veterinary Pathology or in-house at ZV (manual haematology counts and VETSCAN^®^ VS2 Chemistry analyser, Zoetis, NJ, USA).

### 2.3. Data Extracted for Analysis

Data for all species from triage records were extracted, including species, field station, outcome at 24 h after presentation, and evidence of direct (e.g., burn lesions) or indirect (e.g., dehydration) fire impact. Other fields in the triage records had substantial amounts of missing data for many species and were not suitable for analysis.

Further detailed investigation was possible for koalas due to more comprehensive field station and hospital records; therefore, additional data was extracted. Triage records and ZIMS medical records for all koalas were reviewed and collated by one of the coauthors (BM, an experienced wildlife veterinarian) in preparation for analysis. Due to numerous clinicians being involved in the treatment of these patients, the terminology used in both the triage and hospital records varied. This terminology was reviewed, interpreted and coded by BM to facilitate analysis.

#### 2.3.1. Koala Field Station Record Data

Data extracted from koala triage records included: koala ID; field station where koala was received; date of presentation; date of first examination; sex; TWC as a proxy for age; BCS using the first recorded BCS assessment following presentation, rated 1 to 5 where 1 = emaciated and 5 = excellent [7]; demeanour; hydration status; summary of burn injuries by body region (head, torso, limbs); detailed records of burn distribution including parts affected within the body regions of head (ears, eyes, nares, mouth, other), torso (pouch, genitals, other) and limbs (which limb, number of paws burned including affected digits and palms); burn severity as described below; heart rate; rectal temperature; respiration rate; comorbidities; treatments given including type, route and date(s) of administration for fluid therapy, analgesia, antibiotics and other medications; number of days koala received care at field station; transfer details where relevant including date and receiving authorised shelter or facility; and final outcome for koalas not transferred to ZV.

Records of burn severity required cleaning and interpretation prior to analysis due to the nature of clinician burn recording in koalas during field assessments as follows. Treatment of wildlife in field stations was guided by the Victorian Response Plan for Wildlife Impacted by Fire [11], which outlines terminology describing ‘superficial burns’, ‘partial thickness burns’ and ‘full thickness burns’. However, the 2018 Response Plan document does not provide guidance on objective assessment or grading of burns. Consequently, a variety of descriptive terms (sloughing, singed, swollen, bone exposed, no pain sensation, pink, erythema, degloving, perfect), ranking terms (severe, bad, moderate, minor, mild, slight), and burn severity terminology (superficial, partial thickness, full thickness, first degree, second degree and third degree) were recorded in the koala triage records of burn assessments. Due to the lack of an objective assessment approach and the absence of histological data to determine true burn severity for the triage cases for analysis, these burn assessments were categorised into four main ‘burn score’ categories (and two intermediate categories) as outlined in Table 1. Intermediate scores were used when the majority of burns fitted the lower score category, but at least one burn had a descriptor of the greater score category. The burn score categories approximately map to the ‘superficial burns’, ‘partial thickness burns’ and ‘full thickness burns’ described by Fowler [12] and used by Dunstan et al. [10]. However, we choose not to use these terms to avoid implying certainty of the true histological nature of these burns. For koalas subsequently transferred to ZV wildlife hospitals, the burn scores derived from triage records were verified against records of subsequent observations recorded during hospitalisation to increase confidence in the categorisation process.

#### 2.3.2. Koala Hospitalisation Record Data

Data extracted from koala ZIMS hospital records included: koala ID for data linkage to triage data; burn score categorised as per Table 1; recorded comorbidities; initial subjective veterinary assessment of prognosis; details of treatments given (fluid therapy, analgesia, antibiotics, and other, as for triage data described in Section 2.3.1); clinical observations of demeanour, appetite, water intake, urine output, BCS, weight, hydration status, faecal output, and abdominal palpation findings; observation of burns (healing and/or progression); bandage/dressing changes and associated sedation/anaesthesia; and outcome at six months after initial presentation (died, euthanised, released, still in care). Where relevant data was available at multiple timepoints, all values were extracted with associated dates. There was missing data across a number of fields for some koalas, largely associated with the nature and duration of treatment of these cases.

In addition, clinical pathology parameters were extracted for all testing undertaken during each koala’s hospitalisation. The tests performed and repetition of tests varied between cases based on their clinical management, so not all listed parameters were available for all koalas and the timepoints when testing occurred varied relative to initial presentation or date of transfer. The extracted clinical pathology records included: haematology—red blood cell count (×10^12^ cells/L), haemoglobin (g/L), haematocrit (HCT) (L/L), mean corpuscle volume (MCV) (fL), mean corpuscle haemoglobin (MCH) (fmol), mean corpuscle haemoglobin concentration (MCHC) (g/L), platelet counts (×10^12^ cells/L), white blood cell count (×10^9^ cells/L), leukocyte differential (% including lymphocytes, monocytes, neutrophils, bands, eosinophils, basophils), lymphocyte count (×10^9^ cells/L), monocyte count (×10^9^ cells/L), neutrophils count (×10^9^ cells/L), band count (×10^9^ cells/L), eosinophil count (×10^9^ cells/L), basophil count (×10^9^ cells/L); serum biochemistry—sodium (mmol/L), potassium (mmol/L), chloride (mmol/L), total CO_2_ (mmol/L); Anion Gap (mmol/L); calcium (mmol/L); phosphate (mmol/L); ionised calcium (mmol/L), magnesium (mmol/L), glucose (mmol/L), blood urea nitrogen (mmol/L), creatinine (mmol/L), alanine aminotransferase (ALT) (U/L), aspartate aminotransferase (AST) (U/L), alkaline phosphatase (ALP) (U/L), gamma-glutamyl transferase (GGT) (U/L), amylase (U/L), creatinine kinase (U/L), total bilirubin (µmol/L), albumin (g/L), globulin (g/L), total protein (g/L), cholesterol (mmol/L); serum blood gas analysis—arterial pO_2_ (kPa), O_2_ saturation, pCO_2_ (kPa), HCO_3_ (mmol/L), arterial pH, base excess (mmol/L); and urinalysis—specific gravity, nitrite test strip, and urine test strip for pH, glucose, bilirubin, urobilinogen, ketones, protein, blood, and leukocytes.

### 2.4. Statistical Analysis

The extracted and collated data for all koalas treated, including unique identifiers for each koala, were imported into R statistical software v4.1.1 [13] for cleaning and all analyses.

Initial review of records for all koalas presented to the field stations identified a substantial population (64 koalas) that were likely to have been presented as part of a known active capture and relocation operation conducted in the same fire affected areas due to loss of vegetation (habitat and food source) in bushfire-affected areas. Several criteria were used to identify these koalas: (1) presented at Bairnsdale or Gelantipy on or after 8 February 2020 (several weeks after the fires occurred), (2) BCS ≥ 3, (3) no recorded observations of fire injury, (4) no treatment administered at field station, and (5) released following examination. These koala records were created as part of koala assessments by veterinarians at the field stations, but they were considered not to be part of the population of bushfire-affected koalas informing this study and were therefore excluded from analyses. A further six koalas did not have a clear final outcome in records and were also excluded from univariable and multivariable analyses.

All data were cleaned and triage data and hospitalisation data linked using koala ID. Final outcomes for each koala at 6 months post-presentation (or latest date before 6 months for which outcome data was available) was collated from triage and hospitalisation records in four categories: released, in care, euthanised or died. A binary survival outcome variable was then derived from these data, defined as whether the koala had a ‘positive outcome’ (released or in care at 6 months) or ‘negative outcome’ (euthanised or died). Date of transfer to ZV wildlife hospital was simplified to a binary yes/no variable to indicate whether transfer had occurred. Day of presentation was calculated from presentation date, defined as the number of days after 1 January 2020 (when the first field station became operational). Ten koalas had their age recorded only as ‘adult’ and were re-categorised as TWC III for analysis based on feedback from treating clinicians (pers. comm.). Free text fields describing treatments including antibiotics, cissipride and analgesia were collapsed into binary yes/no variables. Burn score was categorised as an ordinal variable as per Table 1. A binary burn severity variable was then derived, defined as minor burns only (grade 1) vs. worse than minor burns (grade ≥ 1.5). The binary burn severity variable was used for simplicity during exploratory analyses, with the ordinal burn score variable used to further understand any associations identified during multivariable analyses of outcomes. Burn distribution records were used to calculate the estimated body surface area affected for koalas with burn injuries, using the body surface area proportions described in Eddy et al. [14].

To reflect variation in the clinical condition and treatment of the koalas, analyses were undertaken for koalas grouped in three ways: (1) all koalas assessed at the field stations (apart from those excluded as described above); (2) koalas with recorded burn injuries; and (3) koalas transferred to ZV for further treatment. These subsets were not independent, but were each reviewed separately to enable evaluation of different clinical factors for association with outcomes. In each subset, data was initially reviewed descriptively. Univariable analyses were then undertaken to assess associations between possible risk factors available in the data and the two key binary outcome variables: burn score and survival outcome. For the koalas transferred to ZV, descriptive and univariable analyses of all clinical pathology parameters were undertaken to attempt to identify specific laboratory results associated with either outcome. Chi-square and Fisher’s exact tests were used to evaluate univariable associations, with test selection based on contingency table frequencies.

To further investigate the interplay of factors associated with koala outcomes in univariable analyses for (1) all koalas assessed and (2) koalas with burn injuries, multivariable analyses were undertaken using the binary survival variable as the outcome of interest, with two generalised linear models using logit link functions (i.e., logistic regression models) constructed using a backwards stepwise approach. The ‘assessed koalas’ multivariable model initially included koala sex, presence of burn injuries and dehydration of 5% or greater, but these did not significantly improve model fit. The final ‘assessed koalas’ model included three explanatory variables: day of presentation (numeric), TWC (ordinal data I, II, III, IVa, IVb, IVc, modelled as numeric), and BCS (ordinal, modelled as numeric, centred at 3). The ‘burnt koalas’ multivariable model initially included day of presentation, sex and presence of dorsal burns on the paws, but these did not significantly improve model fit. The final ‘burnt koalas’ model included four variables: TWC and BCS (both numeric as described for the first model), as well as burn severity (modelled as a binary categorical variable, contrasting burn score = 1 as reference with burn score ≥ 1.5) and the number of digits burnt (categorical with three groups, 0 digits burnt, 1 to 10 digits burnt, or 11 to 20 digits burnt, modelled using dummy variables where 1 to 10 digits burnt was the reference group). For both models, model assumptions were tested and predictor variables were found to be linear in the logit with no influential observations or multicollinearity detected.

To increase the utility of the results of these multivariable analyses, further modelling of the data for koalas with burn injuries was undertaken using classification and regression tree (CART) models. In a CART model [15], data are split one predictor variable at a time using recursive partitioning, and prediction models are fitted and compared for each partition to determine the optimal split [16]. While several algorithms are available for this process, this study used the RPART algorithm [17] to determine the variable and values for each split, so that the two resulting groups for each split have the greatest similarity within groups and greatest difference between groups in terms of prognosis (i.e., with regard to the binary survival outcome). This process of splitting data by individual variables is repeated within each branch of the developing tree, until a single optimal tree is produced. The CART approach is useful in datasets such as this study when the data may contain complex non-linear interaction terms, is more flexible than conventional regression techniques and is more straightforward for clinical interpretation and communicating through visualising the final tree. A CART model was constructed for the subset of koalas with burn injuries, with all variables recorded for this population available for inclusion during model development.

In both modelling approaches, it is important to note that the results presented do not imply a causal relationship between the predicting factors and the survival outcome of interest. There may be complex biological factors influencing survival that are not fully captured in the dataset analysed in this study. Rather, these results can be interpreted as indicating the patterns of association between risk factors and outcomes as observed in this population of koalas, many of which are biologically plausible. It is expected that the results presented here, particularly the visualisations of the CART model, will be used in combination with clinical judgement in determining the likely prognosis of any individual koala receiving veterinary treatment following exposure to bushfires.

## 3. Results

### 3.1. Overall Outcomes for All Animals Presenting to Field Stations

At the field stations in the period 1 January 2020 to 21 February 2020, a total of 259 animals were presented and assessed, including 35 unique species. The majority were koalas (77%, *n* = 196), with other presented species including eastern grey kangaroos (*Macropus giganteus*, *n* = 12), grey-headed flying foxes (*Pteropus poliocephalus*, *n* = 7), feathertail gliders (*Acrobates pygmaeus*, *n* = 5), and a range of other species (*n* = 39, details in Table S1). The Mallacoota field station received 101 animals, 149 were presented at Bairnsdale (including Gelantipy), and nine presented at Corryong.

**Figure 2:**
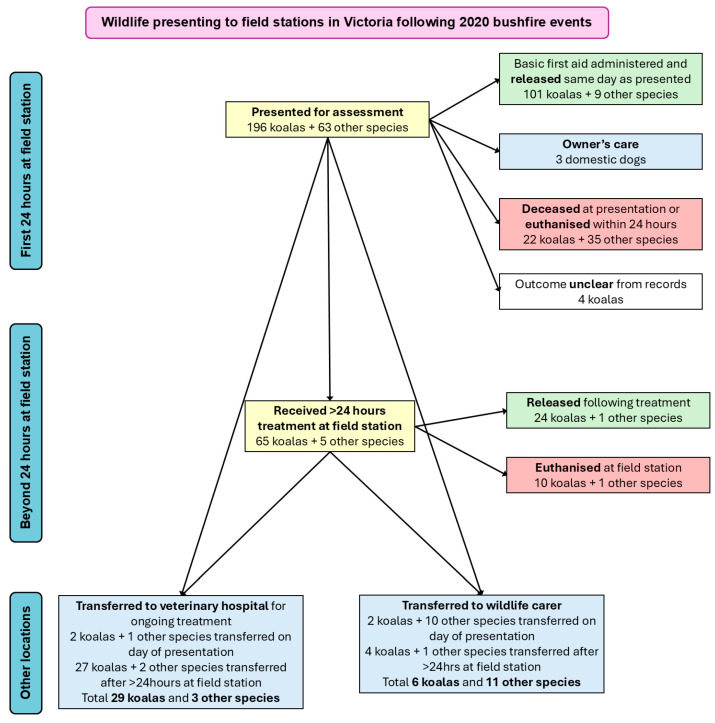
Flow chart of destinations of animals presented to field triage stations during the 2019–2020 bushfire season in Victoria, Australia. Final outcomes following transfer to ZV wildlife hospitals and authorised wildlife shelters are not shown.

Of the 259 animals presented, almost half (47%, *n* = 122) required significant veterinary intervention beyond basic first aid, either euthanasia or ongoing treatment for greater than 24 h. A further 18 animals were transferred to the care of others (veterinary hospitals, authorised wildlife shelters, or in the case of three domestic dogs, their owners) within 24 h. Many of the other wildlife presented for assessment (42%, *n* = 110) were able to be released within 24 h after initial examination, including 64 koalas presented as part of capture and relocation activities due to habitat destruction by fire (as described in methods). Of the remaining animals, five were dead on arrival or died shortly after presentation, and the management undertaken and outcomes for four animals were unclear from the records available. An overview of the initial treatment of all animals presented is shown in Figure 2, with more details provided in Table S2.

Almost half of the animals presented (126/259, 49%) were assessed as being affected by fire, with 104 (40%) having direct signs of impact, including burn injuries, fur with singes or notable soot coverage. In a further 22 (8%), the sole physical sign of fire impact was clinically significant dehydration. Of these bushfire-affected animals, 79% (100) were koalas, with burns, singes or soot noted in 86, and dehydration in a further 14. Of 12 kangaroos presented, ten showed direct fire injury, with burns primarily located on the hindlimbs. One kangaroo also had forelimb burns and one had singed ears and ocular discharge. A full breakdown of the species presented with fire impacts is provided in Table S3.

At 24 h following initial presentation, 85 animals (33% of animals presented) required ongoing veterinary treatment, including 69 koalas. The majority of these animals were treated on-site at triage stations for up to 8 days, largely due to limited transport options associated with fire impacts and related road closures. Smaller numbers of animals were able to be immediately transferred to either ZV (*n* = 3) or authorised wildlife shelters (*n* = 12). Of the 69 koalas receiving treatment, 42% (*n* = 29) went on to be transferred for medical care at ZV wildlife hospitals, described in more detail in Section 3.4.

### 3.2. Clinical Outcomes for All Koalas Presented for Assessment

There were 126 rescued koalas presented for treatment that met the inclusion criteria for this study, of which 52% were released (*n* = 66), 33% died or were euthanised (*n* = 42), and the remaining 18 animals were still in care 6 months after initial presentation. Field stations were first established in early January 2020, and koalas were steadily presented throughout January and into February (see Table 2). Negative outcomes were most common in animals presented in the first two weeks of field station operations. In the initial week, only 29% (5/17) of koalas presented survived, despite being predominantly young animals (TWC ≤ III). The second week was characterised by a higher proportion of aged koalas (18/48 koalas presented were in TWC ≥ IVa), with an overall survival of 65%. In the group of koalas presented in the second week with negative final outcomes, aged koalas were overrepresented (13/17, 76% animals who died or were euthanised). Survival was more consistent for animals presented from the third week onwards, with more than three quarters of presented koalas surviving, and approximately three quarters of all negative outcomes occurring in aged koalas. Two thirds of koalas presented continued to be aged animals (TWC ≥ IVa).

First, we present results of univariable analysis for all assessed koalas, undertaken to better understand factors associated with clinical outcomes. Of the 126 koalas presented, 115 had data for age and sex recorded. Ten koalas were missing TWC data, one had missing sex data, and one was missing both TWC and sex. Two thirds (67%, *n* = 76) were in TWC I, II or III, including 39 females (65% of all female koalas presented) and 37 males (68% of all male koalas presented). The probability of transfer for ongoing care to a ZV wildlife hospital was not associated with TWC (*p* = 0.60), with transfer occurring for 29% of aged koalas (TWC ≥ IVa) compared with 27% of non-aged koalas (TWC ≤ III). Slightly more male koalas (32% of all male koalas presented) were transferred compared to females (25% of all females presented) but this difference was also not statistically significant (*p* = 0.85).

Survival was significantly less likely in older koalas (with TWC ≥ IVa) presented for treatment compared with koalas with TWC ≤ III (*p* < 0.001). Low BCS was also associated with negative outcomes (*p* = 0.014), with 59% survival for koalas in BCS ≤ 2.5 compared with 83% for koalas in BCS ≥ 3. There was no significant difference in survival (*p* = 0.065) for male koalas (59% released or in care at six months) compared with females (74% released or in care at six months). BCS was not associated with koala TWC or sex (*p* = 0.26 and *p* = 0.67, respectively). Of the three field stations, koalas presented to Mallacoota had significantly more negative outcomes (56% survival, 33/59) than Bairnsdale (78% survival, 45/58, *p* = 0.013), while Gelantipy had intermediate survival for the small number of koalas included in this analysis (66% survival, 6/9). There was no significant difference in the proportion of koalas with TWC ≥ IVa presented to field stations (Bairnsdale compared to Mallacoota *p* = 0.889 and Gelantipy *p* = 0.940). The koalas presented at Mallacoota were significantly more likely to have burn injuries (compared to Bairnsdale *p* = 0.015), and were presented early in triage station operations, with 90% of koalas at Mallacoota (53/59) received in the first three weeks of January 2020. There was a significant association between ≥5% dehydration and negative outcomes (*p* = 0.015), but the interpretation of this finding was complicated by concurrent associations between dehydration and aged koalas (*p* = 0.034), low BCS (*p* < 0.01) and the presence of fire injury (*p* < 0.01).

In addition, we present multivariable ‘assessed koalas’ results, to understand the interplay of the factors described above with survival. In this analysis, 37 koalas were excluded due to missing data for age or BCS, therefore records for 89 koalas were evaluated. Negative outcomes were significantly more likely for koalas presented sooner after the fire, older koalas (greater TWC) and koalas with lower BCS. For each week after the initial establishment of the field stations a koala was presented, the odds of a negative outcome were reduced by one third (OR per day 0.94). A one-class increase in TWC (e.g., III to IVa, IVa to IVb, and so on) was associated with more than twice the odds of a negative outcome (OR 2.70). Each decreasing point of condition score was associated with a substantial increase in the odds of a negative outcome (OR 7.27). Of all koalas presented to the field stations (including burnt and non-burnt koalas), the greatest odds of survival (release or in care at 6 months) were associated with young koalas in high BCS presented some time following the initial fire event. Detailed results are presented in Table 3.

### 3.3. Clinical Outcomes for Burnt Koalas

To better understand factors associated with survival for koalas with burn injuries, we present univariable analyses for the subset of 83 koalas with burn injuries and documented 6-month outcomes (66% of the 126 koalas evaluated). Similar to the results for assessed koalas reported in Section 3.2, age was associated with survival with negative outcomes more likely in burnt animals with TWC ≥ IVa (12/30 survived) compared with TWC ≤ III (32/46 survived, *p* = 0.01). Male koalas with burn injuries were more likely to experience negative outcomes compared with female koalas (18/39 or 46% of males with burn injuries survived, compared to 29/42 or 69% of females surviving, *p* = 0.037). A significant effect of BCS was not demonstrated in this smaller subset of burnt koalas (BCS ≤ 2.5 survival 55% compared to BCS ≥ 3 survival 77%, *p* = 0.07) despite the numeric decrease in survival of lower BCS koalas evident.

When burn severity was contrasted between grade 1 only compared with grade 1.5 to 3, a clear difference in outcomes was observed with negative outcomes more common in koalas with higher grade burns (*p* < 0.001). Koalas with grade 1 burns survived in 85% of cases (35/41), compared with just 31% (13/42) survival in koalas with at least one burn recorded as grade 1.5 to 3. Furthermore, these outcomes differed between age groups based on TWC; six koalas with negative outcomes despite having only grade 1 burns were all in TWC ≥ IVa. However, there was not an overall association between burn severity and TWC (*p* = 0.77).

A clear biological gradient was present: the more severe the burns were, the more likely a negative outcome occurred (details in Table 4). Compared with koalas with grade 1 burns only, koalas with burns that were grade 2, grade 2.5 or grade 3 were significantly more likely to have a negative outcome (*p* = 0.010, 0.015, and <0.001, respectively).

The total body surface area burnt for koalas as recorded in the clinical data ranged from <1% to 13.1%, in most cases confined to affect the feet, face and ears. The most common location for burns was the feet, with 90% (*n* = 75) of burnt koalas having burns to one or more feet (with a burnt foot defined as having one or more digits burnt, with or without a burn to the palm and/or dorsal surface of the foot). The most common burn pattern was four feet burnt (*n* = 41); however, these koalas did not have significantly different outcomes compared with koalas with fewer feet burnt or no feet burns (*p*-values ranged from 0.12 to 1.0). Even after adjusting for burn severity, there was still no significant difference in outcomes associated with the number of feet burnt. One quarter (21/83) of koalas had burns to one or more body areas other than the feet, but these koalas did not experience significantly different outcomes compared with those with feet burns only (*p* = 0.27). There was, however, a subset of six burnt koalas who all had negative outcomes. These were in TWC ≤ III with grade 1.5 or higher burns to four or more discrete body areas in addition to feet burns.

There were 46 koalas with burns to the palmar/plantar surfaces of one or more feet, including nine that also had one or more dorsal surface burns. Six of the nine koalas with dorsal feet burns had all four feet burnt on both dorsal and palmar/plantar surfaces, and five of these had grade 1.5 or higher burns. Eight of the koalas with dorsal feet burns were euthanised at the field stations, with only one transferred for intensive veterinary care (including two months of burn dressings applied) and still in care 6 months after initial presentation. For koalas with palmar/plantar foot burns without dorsal foot involvement, survival was highest in those with grade 1 burns only (9/12 survived, 75%) compared with those having grade 1.5 or higher burns (9/21 survived, 43%); however, this difference was not statistically significant (*p* = 0.09).

**Figure 3:**
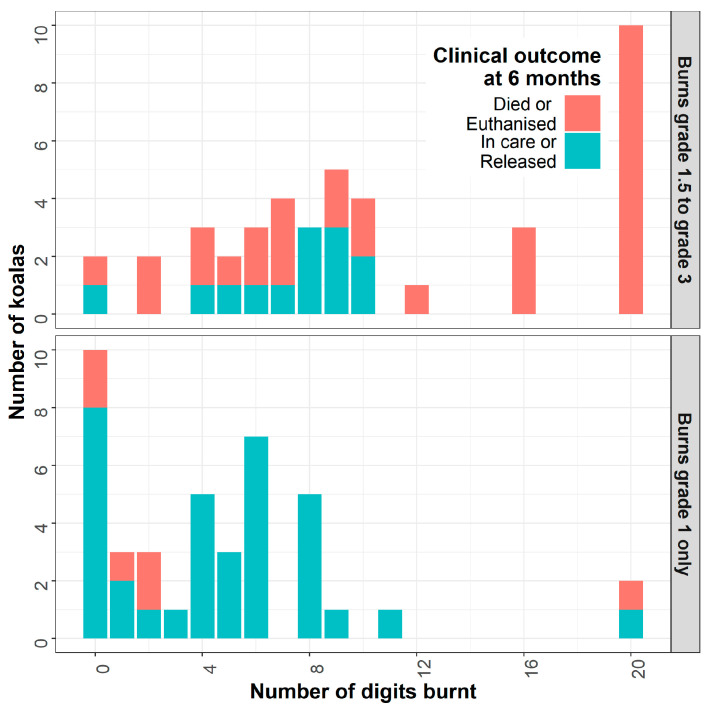
Clinical outcomes for 83 koalas with burn injuries from bushfire-affected areas presented to field stations in 2020 in Victoria, Australia, stratified by number of digits burnt and burn severity. Burn score for each koala is categorised as ‘grade 1’ = minor burns only or ‘grade 1.5 to grade 3’ = at least one burn worse than minor.

Although the number of feet burnt was not associated with survival outcomes for koalas, there was a significant association of outcomes with the pattern of burns to the digits. Koalas with no digital burns had similar outcomes (9/12 survived, 75%) to koalas with burn injuries to ≤10 digits (37/54 survived, 69% survival, *p* = 0.074). However, koalas with ≥11 digits burnt were much more likely to have a negative outcome (12% survival, 2/17, *p* < 0.001 compared with koalas with ≤10 digits burnt). Twelve koalas had all 20 digits burned, and all but one of these had a negative outcome. Digital burns that were worse than mild were also associated with poorer outcomes (visualised in Figure 3), especially in koalas with ≥11 digits burnt. Of 40 koalas with digital burns scored as grade 1.5 or higher, 28 (70%) had negative outcomes, with 100% negative outcomes in koalas with more than 10 digits with these higher grade burns. This is compared with koalas with grade 1 digital burns to one or more digits, where only 4/31 (13%) had negative outcomes. Only three koalas with grade 1 digital burns had more than 10 digits burnt, of which two survived. In these koalas with superficial digital burns, the number of digits affected was not strongly associated with outcomes. There were no significant differences in outcomes associated with digital burns on the front versus rear feet.

To further understand how factors combine to affect survival in burnt koalas, we present multivariable ‘burnt koalas’ results based on analysis of 61 burnt koalas (Table 5). In total, 22 koalas were excluded due to missing data for age or BCS. Negative outcomes were significantly more likely for older koalas (OR 5.11 for each one-class increase in TWC) and koalas with lower BCS (OR 6.67 for each one-unit decrease in BCS), mirroring the results found for rescued koalas including both burnt and non-burnt koalas. In addition, overall burn severity for each koala and the number of digits burnt were associated with outcomes, after adjusting for the other variables in the model. Koalas with burns scored as grade 1.5 or higher had ten times the odds of a negative outcome compared with koalas with grade 1 burns only (OR 10.23, 95% CI 1.36 to 147.74, *p* = 0.043), and koalas with 11 or more digits burnt had markedly increased odds of a negative outcome compared with koalas with 10 or fewer digits burnt (*p* = 0.007, OR > 100, 95% CI 8.84 to >1000). Koalas with no burnt digits had similar odds of a negative outcome compared with those with 1–10 digits burnt.

**Figure 4:**
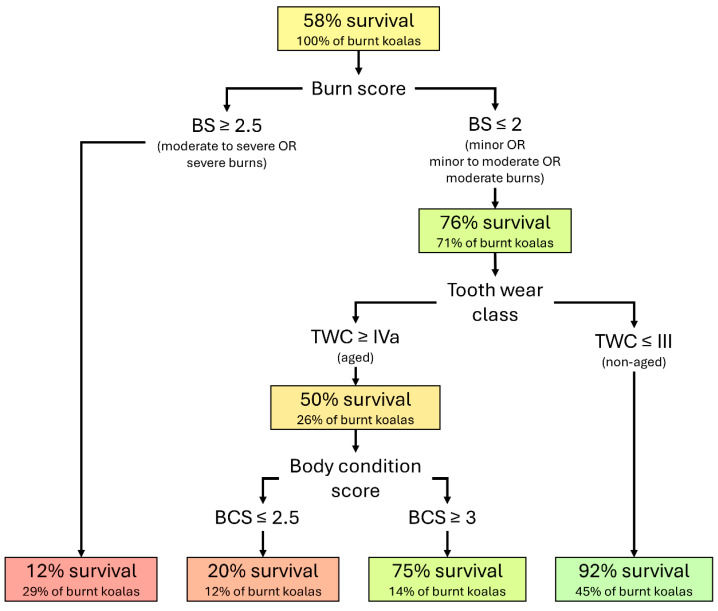
Classification tree model for survival outcomes for 83 (100%) bushfire-impacted koalas with burn injuries with overall 58% survival. Each node of the tree is a binary decision point for survival for a single factor (such as burn score), with each ‘branch’ of the tree representing two possible values of that factor (for burn score, BS ≥ 2.5 or not) that are associated with survival outcomes. The underlying model splits the preceding group of koalas at this single value of the factor of interest, to maximise the difference in percentage survival between the split groups. Box for each branch shows percentage survival and percentage of all burnt koalas represented; colours show the gradient of survival outcomes, with green = majority survival and red = majority non-survival.

To increase the utility of these results for clinicians for decision making, a classification and regression tree (CART) model for the same 61 ‘burnt koalas’ records is presented (Figure 4). Based on the underlying data, the poorest outcomes were observed in koalas with grade 2.5 or grade 3 burns, with only 12% of these koalas surviving independent of all other factors. For koalas with burns of grade 2 or less, young koalas had the best outcomes, with 92% survival in these koalas where TWC was III or less. For older koalas with TWC ≥ IVa, there was a notable difference in survival based on the BCS of the koala; 75% of koalas in BCS ≥ 3 survived, compared with only 20% survival in koalas with BCS ≤ 2.5.

### 3.4. Clinical Treatment of Hospitalised Koalas

There were 29 koalas transferred from the Mallacoota and Bairnsdale field stations for treatment at ZV wildlife hospitals, of which 22 had burn injuries and 19 (66%) survived to 6 months after initial presentation. These koalas received a range of intensive treatments, including: intravenous fluids (24/29 koalas, with 15/24 receiving >24 h of fluid therapy); one or more doses of analgesics (19/29 koalas) including painstop, paracetamol, carprofen, burprenorphine, fentanyl or tramadol; two or more instances of bandaging (20/22 koalas with burn injuries); one or more doses of cissipride (12/39 koalas); and one or more doses of antibiotics (14/29 koalas). The combination of treatments received by any one koala was based on the clinical judgement of the treating clinicians, with further narrative details of treatment available in Parrott et al. [1].

Univariable ‘transferred koalas’ results are presented to allow comparison between this group of animals and the broader groups presented above. Similar to Section 3.2 and Section 3.3, TWC was associated with clinical outcomes in the transferred koalas. There were 11 transferred koalas with TWC ≥ IVa and this group was significantly more likely to experience a negative outcome compared to 18 TWC ≤ III koalas (OR = 7.972, 95% CI 1.181 to 72.663, *p* = 0.0169). Outcomes for the transferred koalas were not significantly different between sexes (*p* = 0.128), BCS groups (*p* = 1.0) or which field station koalas were transferred from (*p* = 1.0). There was no clear association between the week these koalas were first presented to the field station and their outcomes. The number of days elapsed before transfer from a field station to a ZV wildlife hospital was mediated by the need to stabilise the koalas as well as the availability of transport out of the bushfire-affected region where the field station was located. Transfer occurred between zero days (i.e., day of presentation) to seven days after initial presentation, with a median of three days, and there was no clear pattern of outcomes associated with the number of days elapsed prior to transfer. The clinical features of each case, rather than human-controlled factors around presentation and transfer dates, appears to best explain the outcomes for the transferred koalas.

Burn patterns for the transferred koalas varied and were associated with final outcomes. Of the 22 koalas with burn injuries, 14 (64%) survived. All 22 had burns to one or more feet and seven also had burns to other anatomical areas. Based on the patterns of burns in the koalas’ clinical records, it is very unlikely that any of these animals had >10% of their body surface area burnt. There were seven koalas that had ear burns that were not detected at initial presentation but were identified a median of 5 days later (range two to 15 days post-presentation). Although these delayed ear burns were not associated with outcomes, they should be noted for their relevance to clinical assessment and management of koalas with burn injuries.

There were 14 koalas with burns to all four feet, and a further five koalas with three feet burnt. All burns not involving the feet were grade 1, but the burns to feet ranged from grade 1 to grade 3. Of koalas with burns to all four feet, 10/14 survived, including all koalas with grade 1 feet burns. Of koalas with burns to three feet, 2/5 survived, both of which had grade 1 burns. Four koalas had grade 1 burns to the palmar/plantar surfaces of the feet, and ten koalas had grade 1.5 or higher palmar/plantar burns. The number of digits burned ranged from 0 to 20, with a median of seven digits burnt. Negative outcomes were more common in the transferred koalas with grade 2.5 or grade 3 digital burns (3/8 or 38% survived with a range of 2 to 20 digits burnt). Only one survived (17%) of six koalas where all five digits on one or more paws had burns of grade 1.5 or higher. The small number of cases in the transferred group prohibited drawing stronger conclusions about digital burns in this group, apart from a broad pattern in the data that koalas with combinations of higher grade burns and more digits affected often had poorer outcomes than koalas with fewer, low grade digital burns. There were no appreciable differences in outcomes for burns of forelimb feet and digits compared with the hindlimbs.

In reviewing the clinical pathology results for these 29 koalas across their periods of hospitalisation, very few had parameters that were outside the validated laboratory reference ranges provided with their results. No clinical pathology parameter showed a significant, consistent association with burn grade or 6-month outcomes.

Of 14/29 (48%) koalas that received antibiotics during their period of hospitalisation, seven survived. There was no significant difference in survival between transferred koalas that did or did not receive antibiotics (*p* = 0.13). For koalas in TWC ≤ III, survival outcomes were the same in koalas that received antibiotics (5/6 survived, 83%) and those that did not (10/12 survived, 83%). A difference was appreciable between older koalas (2/8 receiving antibiotics survived, compared with 2/3 surviving that did not receive antibiotics), but the lack of consistency across the two age groups suggests this may be attributable to differences in the clinical condition of these groups rather than the antibiotics themselves. There was no significant difference in survival associated with the number of days antibiotics were administered for (*p* = 0.26).

Intravenous (IV) fluid therapy was also not associated with differences in survival outcomes, with no significant differences in outcomes between koalas that did or did not receive IV fluid therapy (*p* = 0.63), or between koalas with IV fluids administered for >24 h compared with short-duration or no IV fluids (*p* = 0.22).

## 4. Discussion

This study of wildlife treatment records from bushfire-affected locations in Victoria gives an overview of the species presented along with insights into prognostic indicators associated with survival in koalas. Older koalas, those in lower BCS, and those presented in the first days after fire were more likely to die or be euthanised, independent of the presence of burn injuries. For koalas with burn injuries, similar factors were associated with survival, along with burn severity and the extent of burns to digits. In burnt koalas with low total body surface area affected (BSA <15%, such as those in this study), the specific areas burnt, and the severity of the worst burns appear to be useful indicators of prognosis. This is consistent with Dunstan et al. [10] who emphasise considering both the location and severity of burns, and Baek et al. [18] who call for increased attention to burns in specific anatomical areas such as the feet. Clinician judgement of the severity of injuries and the associated prognosis was also a factor in determining survival, given that most koalas that did not survive were euthanised.

To interpret these results in context, we recognise that decision making for management of injured wildlife is complex and while this study focussed on clinical parameters and likely medical outcomes, there are a host of other factors that also need to be considered when deciding which animals to treat. These include the impact of having wild animals hospitalised for prolonged periods and the resultant stress and restricted behavioural opportunities; loss of fitness, and altered social context when released; the health of the habitat they are going to be released back into and what impact their introduction into this habitat may have on other cohabitating species; the overall economic costs of their rehabilitation; and last, but by no means least, the impacts of tending to these animals for prolonged periods on the mental health of veterinarians and carers, especially if long-standing cases do not have positive post-release outcomes. Even in the presence of positive prognostic signs based on clinical evaluation, at times euthanasia may still be the most humane decision, given that good animal welfare in rehabilitated animals must be more holistic than positive health outcomes alone. However, the aim of this study was to provide clinicians with a more objective understanding of the likely medical prognosis when managing bushfire-affected animals to avoid prolonged treatment in animals unlikely to have a meaningful clinical recovery.

The results of this detailed review of records of koalas presented to triage stations suggest that those most likely to recover from their injuries include young koalas, koalas in good BCS, and koalas with mild burns that do not involve large numbers of digits. These prognostic indicators assume adequate and appropriate veterinary care is provided by those with expertise in koala medicine, and do not apply to severely injured animals that are euthanised immediately following capture on humane grounds. The factors identified in this study, especially the association between outcomes and demographic factors (koala age and BCS) should also inform future data collection during bushfire response, ensuring the right information is gathered to further enhance the evidence base for managing bushfire-affected koalas. In line with Dunstan et al. [10], a standardised format for collecting clinical data to facilitate retrospective analyses of wildlife fire injuries would be extremely valuable.

Further investigation of the koalas that received intensive clinical treatment (having been transferred for hospitalisation at a wildlife-specific veterinary clinic) suggested that again, koala age and the combination of burn severity and the extent of burns to digits were associated with survival. Detailed review of a range of routine clinical pathology parameters measured in these koalas revealed that most parameters were within reference intervals and none were specifically associated with survival, similar to Lane et al. [19]. This contrasts with Funnell et al. [20] who found elevated serum sodium likely related to dehydration at presentation in a cohort of koalas with burns sufficiently severe to require bandaging. This indicates that the koalas selected for transfer were not severely compromised systemically. Our result does not rule out the possibility that other, non-routine clinical pathology tests may be useful for prognostic assessment; a pilot investigation of such tests would be a useful first step. Overall, the data suggest that koala age and the nature of burn injuries should be considered when deciding if a koala is suitable for hospital treatment, with further management informed by the individual animal’s clinical condition rather than using a standardised treatment protocol.

In emergency responses to bushfires, timely and accurate assessment of injured wildlife is critical for objective decision making, to effectively manage the welfare of impacted animals and make the most effective use of the resources and personnel available for the response. In addition, the ability to make these decisions more objectively will also benefit the mental health of attending veterinary staff knowing their investments are likely to manifest more positive outcomes overall. There are few reports evaluating burn injury management and prognosis for wildlife to inform those working in the area [10,21,22], with several specific calls for greater prognostic guidance [10,23]. Existing recommendations have often been anecdotal or extrapolated from companion animal medicine [12]. The detailed records generated by the large number of animals assessed in Victoria in 2020 allowed this study to further describe and evaluate objective measures that may be useful prognostic indicators. While the decision as to whether a koala is suitable for ongoing supportive care and medical management will always rely on the clinical judgement of the assessing clinician, structured evidence can help support these clinical decisions.

Koala age and BCS in the clinical records were determined by experienced ZV staff using established methods. The age and sex distributions of the koalas included in this study are aligned with populations described by McLean [24], suggesting the presented koalas were a cross-section of their source populations (rather than a particular sex or age group being more at risk). Age, measured as TWC, was consistently significantly associated with survival, with poorer outcomes in aged koalas (TWC > IVa). In this study, the koalas that died or were euthanised despite only having superficial burn injuries were all aged koalas. A correlation between age and negative outcomes for bushfire-affected koalas was not reported by either Dunstan et al. [10] or Wallis [25], although this may reflect methods of assessing TWC and/or population structures in those studies. Sex was rarely associated with outcome except in the koalas with burn injuries with males having lower survival, counter to findings by Dunstan et al. [10] in koalas from Kangaroo Island. This suggests koala sex may require further investigation to determine its prognostic relevance.

Koala BCS was associated with survival outcomes throughout our analyses. The effect of BCS on outcome (or on the decision to euthanise koalas) may have a component that is independent of burn injuries; it is common for koalas rescued from fire grounds to have low BCS [19], which may be because they are easier to capture. Furthermore, it would not be surprising if veterinarians were more likely to euthanise koalas presenting in poor body condition, independent of the presence of burn injuries. Nonetheless, a koala in poor BCS with burns in a pattern associated with a poor prognosis likely has an even lower chance of survival. Dunstan et al. [10] reported a strong association between poor body condition and negative outcomes on Kangaroo Island. It is difficult to eliminate the range of potential factors impacting BCS when considering its prognostic significance, so we propose it should be treated as an indicator of general health. As such, it is appropriate to consider BCS in clinical assessment of bushfire-affected koalas in the field.

Dehydration at the time of assessment has been identified as a prognostic indicator in previous work [10] but was not significant in multivariable analyses in this study. While dehydration was associated with negative outcomes during univariable analysis, it was also significantly more likely to be present in koalas with poorer prognosis based on age (older koalas), BCS (lower) and koalas with burns. It is therefore difficult to determine whether the extent of dehydration has a direct impact on prognosis (and should be cause for concern even in young unburnt koalas), or if it is a consequence of these other factors and reflects the individual koalas’ general ability to cope with and recover from bushfire effects, or even the suitability of the habitat it was occupying at the time of the fire event. Nonetheless, this supports ensuring assessing veterinarians are informed by the overall clinical impression of each individual animal, and that prognostic algorithms such as Figure 4 should be used to support rather than replace clinical judgement.

Koalas presented in the earliest days post-bushfire had poor survival. Given presentation to triage stations in later weeks requires an animal to have survived the intervening period, the most severely affected animals are likely to only be seen in the first days (as they will otherwise succumb to their injuries prior to being captured and brought for assessment). This is consistent with Dunstan et al. [10] who reported more positive koala outcomes later in the bushfire response. Many animals required treatment following initial assessment, either in one of the three field stations for varying periods prior to release or in ZV wildlife hospitals after being transferred. There was no clear pattern to indicate that shorter or longer stays in field stations prior to transfer impacted survival, suggesting that field treatment and decisions made around timing of transfers were appropriate and did not compromise outcomes of these patients. In addition, there was no clear pattern between azotaemia and the time animals had spent in the field stations prior to transfer, again supporting that field treatment did not compromise outcomes of these patients. It is likely the biggest benefit of timely transfer was more effective management of the workload of veterinary teams involved and reduced work in transporting necessary consumables (such as medications, bandage materials and IV fluids) to triage stations.

Consistent, repeatable classification of burn severity in wildlife is not well established, presenting an ongoing challenge. Similar to Dunstan et al. [10], we retrospectively classified burn severity based on the clinical classifications and descriptions in records, and recognise the limitations associated with this approach, notably that clinical examination does not always reveal the true severity of burns, especially in the first days following burn injury [18]. Nonetheless, in the absence of histological evaluation of individual koala’s burns in this study, based on clinical descriptions, we categorised the burns into minor (grade 1), moderate (grade 2) and severe (grade 3), with two intermediate groups where animals had multiple burn severities at different anatomical sites recorded. ‘Moderate’ and ‘severe’ categories approximate to ‘partial thickness’ and ‘full thickness’ burns in Dunstan et al. [10], but we avoided these terms given the true extent of tissue damage could not be determined from the records reviewed. Given the limitations of the classification approach, and the clear biological gradient observed in our data (more severe burns associated with poorer outcomes), we collapsed our five-point grading system into two categories for further analysis: grade 1 only or grade 1.5 to 3. A clear difference in outcomes was apparent with this binary categorisation. While clearer guidelines on assessing and recording burn severity will need to be developed to assist with triage assessments in the future, this simpler categorisation may facilitate rapid field assessment of burn severity and associated prognosis.

For koalas, feet were the most common site burnt, with the most common pattern of injury being all four feet affected, consistent with previous reports in this species [10,18,20,21,26]. There was no clear association between the number of feet burnt and prognosis in this study, even after accounting for burn severity. This differs from Funnell et al. [20] who did find such an association within the subset of most severely burnt koalas (those with feet requiring bandaging). However, in this study the extent of burns involving the digits was associated with outcomes: koalas with burns to more than 10 digits had a far greater odds of dying or being euthanised. These koalas with widespread digital burns often also had extensive paw burns and were typically euthanised following presentation, due to either clinical assessment of the burns or concurrent systemic compromise. Detailed analysis of the number of toes burnt is not presented in Funnell et al. [20] and this may explain the apparent discordance between their results and ours, which likely both reflect the effect of extensive digital burns. Due to the nature of the records reviewed, this analysis did not differentiate between burns to digits and claw damage. However, claw damage and claw loss is a further important feature of severe burns to feet and digits [10,18,20].

If outcomes for the burnt koalas in this study are representative of burnt koalas more generally, overall burn severity and the involvement of digits may serve as useful prognostic indicators. This builds on the findings of Dunstan et al. [10] but suggests a more specific focus on the extent of burns within the feet such as the number of digits affected rather than simply counting the number of feet or body areas affected. In multivariable analysis, digital burns appeared in the regression model but not the classification tree model, being located further down the ‘tree’ (i.e., explaining less of the variance in the data) than the other factors presented. Nonetheless, in a koala with otherwise positive prognostic factors (i.e., a young koala in good BCS presented some days after the actual fire), the presence of widespread grade 1 burns to many digits or more severe burns to a smaller number of digits should motivate deeper consideration by treating clinicians. Validation of the classification tree model (Figure 4) with data from koalas burnt and treated in future fires could help verify the appropriateness of these proposed prognostic factors. As no koalas in this study had extensive burns to greater than 15% of their body surface area, the prognosis for koalas with larger areas of their body burnt may not follow the patterns we have reported.

Three key considerations in the intensive veterinary treatment of koalas from bushfire-affected locations include the use of antibiotics, pain relief and IV fluid therapy. This study reviewed the use of all three treatments in the 29 koalas transferred to ZV. Almost half received antibiotics, with no overall difference in outcomes between these koalas and koalas that did not receive antibiotics, supporting the use of antibiotics in bushfire-affected koalas when clinically indicated. For further evaluation of antibiotic use during the treatment of a larger population of bushfire-affected koalas, we refer to McDougall et al. [27]. A variety of pain relief treatments were administered to the small number of hospitalised koalas in this study, precluding detailed analysis of efficacy. However, given calls for bushfire-affected koalas to be treated and rehabilitated [19], the welfare of these animals during treatment must be prioritised, including provision of appropriate analgesia and decision making by appropriately experienced wildlife veterinary teams in a wildlife hospital. Clinical judgement and response to treatment remain the most useful guide for selection of pain relief in these cases. Administration of IVFT was not associated with survival, suggesting that fluid replacement regimens for koalas at ZV wildlife hospitals were clinically appropriate. In human burns patients, IVFT is indicated where total BSA burnt > 15% [28], and poor prognosis in koalas where burns affected >15% BSA has also been noted previously [29]. Using an approach outlined by Eddy et al. [14], no koalas in this study would have exceeded 15% BSA burnt (although the lack of this systematic approach to estimating BSA burnt in koalas in 2020 may have led to some underestimation of total BSA burnt in the clinical data we reviewed). As such, IVFT should be provided in koalas with similar burn patterns to those in this study based on clinician judgement of hydration status and adequacy of oral intake.

Although this study highlights the broad range of species that may be presented during bushfire events (Table S1), koalas were the most frequently presented species, concordant with previous studies [1,10,23]. As outlined by Parrot et al. [1], factors that may contribute to strong koala representation include their relative inability to evade fire and their arboreal nature, as well as a host of human factors including the ability of rescuers to find and capture affected animals, and a general positive societal and media bias toward koalas. Similar factors may account for the relative low numbers of birds and reptiles presented for care in these data, despite representing a far greater proportion of the free-living fauna in the bushfire-affected areas. We recognise that the population of animals and distribution of species presenting to the field stations and reported in this study represent only a small proportion of those impacted and/or killed by the fires.

This study was limited by its retrospective nature and associated variation in the recording of clinical observations at triage and during hospitalisation. In addition, the results are most relevant to animals that are presented for assessment under similar circumstances to the field stations in this study as described by Parrott et al. [1]. While most records in this study were impressively detailed and amenable to classification, we expect that some useful observations from treating clinicians were not captured. In particular, variation in burn evaluation and recording led to challenges when extracting and classifying the data. This would be improved by using clear guidelines and consistent terminology to assess burns in wildlife and particularly in koalas. In addition, despite this study presenting the largest analysed cohort of hospitalised bushfire-affected koalas reported to-date, the limited number of koalas receiving in-hospital treatment whose records could be analysed precluded stronger conclusions being drawn. A similar study investigating a larger cohort of koalas would allow further analysis and insights, although this may require aggregation of several fire seasons which may also complicate interpretation. In addition, qualitative research contemporaneous to the period of intensive hospitalisations could better capture the impressions of treating clinicians, likely providing further insights for prognosis and clinical judgement, and is recommended in future fire seasons where large numbers of koalas are assessed and treated.

## 5. Conclusions

This study gives a comprehensive overview of data from wildlife presented to triage stations during Victoria’s black summer bushfires. We focussed on identifying prognostic factors that can be evaluated at presentation and in early treatment for koalas, a commonly presented species. Age, BCS and time since the fire are useful factors to inform prognostic assessments for koalas presented from bushfire-affected regions. In animals where burns are present and affect less than 10% of the body surface area, attention should also be given to both the severity of burns and the extent of burns to the digits. Where routine clinical pathology parameters are within normal reference ranges for koalas, these results are minimally informative for assessing prognosis. Once a likely prognosis had been determined and a clinical decision made, clinical management and treatment should rely on veterinary assessment of each individual koala, rather than in a standard protocol-based approach. These results are intended to inform and support future bushfire responses, strengthening clinical decision making which can be integrated with broader considerations for determining how to manage fire-injured wildlife. Key areas for further investigation include the association of koala sex with prognosis, qualitative investigation of the impressions of treating clinicians in determining prognosis, and the establishment of clear and objective approaches to score burns in koalas and other wildlife, which would allow both clearer prognostic evaluation and facilitate more uniform record-keeping to support future similar retrospective studies.

## Figures and Tables

**Table 2:** Weekly summary of outcomes for 126 koalas from bushfire-affected areas presented to field stations in 2020 in Victoria, Australia.

Dates Presented	Koalas Presented (Total, n)	Tooth Wear Class			TWC ≤ III (%)	Outcome at 6 Months		6-Month Survival (%)
		≤III	IVa	Missing Data		Died or Euthanised	Released or Transferred	
1 to 7 January	17	14	1	2	93%	12	5	29%
8 to 14 January	48	27	18	3	60%	17	31	65%
15 to 21 January	24	14	8	2	64%	5	19	79%
22 to 28 January	14	9	5	0	64%	3	11	79%
29 January or later	23	12	7	4	63%	5	18	78%

**Table 3:** Results of multivariable logistic regression for factors associated with ‘negative outcomes’ (death or euthanasia by 6 months after initial presentation) for 89 koalas from bushfire-affected areas presented to field stations in 2020 in Victoria, Australia.

Variable	Unit	Coeff	Std Error	Odds Ratio		*p*-Value
				Estimate	95% CI	
Day of presentation ^^^	+1 day	data	data	0.94	0.88 to 0.99	0.029
Tooth wear class	+1 class	−0.065	0.030	2.70	1.67 to 4.96	<0.001
Body condition score	−1 score *	−0.993	0.272	7.27	2.77 to 26.4	<0.001
Intercept		1.984	0.565			<0.001

^^^ Number of days elapsed since 1 January 2020 when first field station was established in Victoria. * Centred at body condition score (BCS) = 3, where BCS =1 is emaciated and BCS = 5 is excellent body condition. BCS results presented as change in survival for a one-unit decrease in BCS.

**Table 4:** Outcomes for 83 koalas with burn injuries from bushfire-affected areas presented to field stations in 2020 in Victoria, Australia, stratified by burn score where grade 1 = minor burns only, grade 2 = majority of burns assessed as moderate and grade 3 = majority of burns assessed as severe.

Burn Score	Number of Koalas			Survival (%)
	Total	Outcome * In Care or Released	Outcome * Died or Euthanised	
Grade 1	41	35	6	85%
Grade 1.5	6	4	2	66%
Grade 2	12	6	6	50%
Grade 2.5	5	2	3	33%
Grade 3	19	1	18	5%
**Total**	**83**	**48**	**35**	**58%**

* Outcome at 6 months after initial presentation to triage station, as recorded in clinical records. Survival % is calculated as the proportion with ‘outcome: in care or released’ divided by the total in each group.

**Table 5:** Results of multivariable logistic regression for factors associated with ‘negative outcomes’ (death or euthanasia by 6 months after initial presentation) for 61 koalas with burn injuries from bushfire-affected areas presented to field stations in 2020 in Victoria, Australia.

Variable	Unit/ Category	Coeff	Std Error	Odds Ratio		*p*-Value
				Estimate	95% CI	
Tooth wear class	+1 class	1.632	0.534	5.11	2.19 to 19.8	0.002
Body condition score	−1 score *	1.897	0.826	6.67	1.72 to 49.8	0.022
Burn score	Grade 1 burns only	2.326	1.150	10.23	1.36 to 147	0.043
	Grade 1.5 to Grade 3 burns	Ref.	-	-	-	-
Number of digits burnt	No digital burns	0.337	1.284	1.40	0.10 to 19.3	0.793
	1–10 digits with burns	Ref.	-	-	-	-
	11–20 digits with burns	5.749	2.138	>100	8.84 to >1000	0.007
Intercept		−10.067	2.908			<0.001

* Centred at body condition score (BCS) = 3, where BCS = 1 is emaciated and BCS = 5 is excellent body condition. BCS results presented as change in survival for a one-unit decrease in BCS. Coeff = coefficient; Ref = reference category; Std error = standard error; CI = confidence interval. AIC = 43.43.

## Data Availability

Restrictions apply to the availability of these data. Data were obtained from Zoos Victoria and should be requested from this organisation directly. Relevant data are presented or summarised in the manuscript and Supplementary Materials.

## Supplementary Materials

The following supporting information can be downloaded at: https://www.mdpi.com/article/10.3390/ani16060944/s1; Table S1. Free-living animal species presented for assessment to field triage stations during the 2019–2020 bushfire season in Victoria, Australia; Table S2. Detailed outcomes for free-living animal species presented for assessment to field triage stations during the 2019–2020 bushfire season in Victoria, Australia; Table S3. Free-living animals with evidence of direct impact of fire (burns, singes to fur or substantial soot present on assessment) or indirect impact through dehydration only, assessed at field triage stations during the 2019–2020 bushfire season in Victoria, Australia.

## Abbreviations

The following abbreviations are used in this manuscript:

OR	Odds ratio
DELWP	Victorian State Government Department of Environment, Land, Water and Planning
DEECA	Victorian State Government Department of Energy, Environment and Climate Action
ZV	Zoos Victoria
BCS	Body condition score
TWC	Tooth wear class
ZIMS	Zoological Information Management System
CART	Classification and regression tree
