# Supplementary material for: Retrospective Analysis of Triage and Hospitalisation Records for Bushfire-Affected Koalas (Phascolarctos cinereus) and Other Wildlife Species from Victoria, Australia, 2019–2020
Source: Animals (Basel). 2026 Mar 17;16(6):944. doi: 10.3390/ani16060944 (PMC13023346; doi:10.3390/ani16060944)
Supplement: Supplementary file 1 [file animals-16-00944-s001.zip › animals-4126424-supplementary.pdf]

# Retrospective Analysis of Triage and Hospitalisation Records for Bushfire-Affected Koalas (*Phascolarctos cinereus*) and Other Wildlife Species from Victoria, Australia, 2019–2020

**Caitlin N. Pfeiffer [1,*], Bonnie McMeekin [2], Lee F. Skerratt [1] and Richard J. Ploeg [1]**

[1] Melbourne Veterinary School, University of Melbourne, Werribee, VIC 3030, Australia; l.skerratt@unimelb.edu.au (L.F.S.); richard.ploeg@csiro.au (R.J.P.)
[2] Zoos Victoria, Parkville, VIC 3052, Australia; bmcmeekin@zoo.org.au
* Correspondence: caitlin.pfeiffer@unimelb.edu.au

## Supplementary Materials

**Table S1.** Free-living animal species presented for assessment to field triage stations during the 2019-2020 bushfire season in Victoria, Australia.

| Species | Scientific name | Total count of species presented | Count by location of field station | | |
|---|---|---|---|---|---|
| | | | Mallacoota | Bairnsdale (including Gelantipy) | Corryong |
| **Southern koala** | *Phascolarctos cinereus* | 196 | 62 | 134 | |
| **Eastern grey kangaroo** | *Macropus giganteus* | 12 | 8 | 0 | 4 |
| **Grey headed flying fox** | *Pteropus poliocephalus* | 7 | 3 | 4 | |
| **Feathertail glider** | *Acrobates pygmaeus* | 5 | 5 | | |
| **Other species** | See below for breakdown by species | 39 | 19 | 11 | 5 |
| **Totals** | | **259** | **97** | **149** | **9** |
| Asian house gecko | *Hemidactylus frenatus* | 1 | | 1 | |
| Australian pelican | *Pelecanus conspicillatus* | 1 | 1 | | |
| Blue-tongued skink | *Tiliqua scincoides* | 1 | | 1 | |
| Australian boobook | *Ninox boobook* | 2 | | 2 | |
| Common bronzewing | *Phaps chalcoptera* | 1 | 1 | | |
| Common brushtail possum | *Trichosurus vulpecula* | 2 | | | |
| Crimson rosella | *Platycercus elegans* | 1 | 1 | | |

| | | | | | |
|---|---|---|---|---|---|
| Domestic dog (presented by owner) | *Canis lupus familiaris* | 3 | 3 | | |
| Eastern bristlebird | *Dasyornis brachypterus* | 1 | 1 | | |
| Short-beaked echidna | *Tachyglossus aculeatus* | 3 | 1 | | 2 |
| Golden bell frog | *Litoria aurelia* | 1 | 1 | | |
| Lace Monitor | *Varanus varius* | 1 | 1 | | |
| Laughing Kookaburra | *Dacelo novaeguineae* | 1 | | 1 | |
| Lesser long-eared bat | *Nyctophilus geoffroyi* | 1 | 1 | | |
| Little shearwater | *Puffinus assimilis* | 1 | 1 | | |
| Eastern long-necked turtle | *Chelodina longicollis* | 2 | | 2 | |
| Musk lorikeet | *Glossopsitta concinna* | 1 | 1 | | |
| New Holland honeyeater | *Phylidonyris novaehollandiae* | 1 | 1 | | |
| Australian owlet-nightjar | *Aegotheles cristatus* | 1 | | 1 | |
| Red-bellied black snake | *Pseudechis porphyriacus* | 1 | 1 | | |
| Red-necked wallaby | *Macropus rufogriseus* | 1 | 1 | | |
| Common ringtail possum | *Pseudocheirus peregrinus* | 1 | 1 | | |
| Sacred kingfisher | *Todiramphus sanctus* | 1 | | | 1 |
| Silver gull | *Chroicocephalus novaehollandiae* | 1 | 1 | | |
| Sugar glider | *Petaurus breviceps breviceps* | 1 | 1 | | |
| Superb lyrebird | *Menura novaehollandiae* | 1 | 1 | | |
| Swamp wallaby | *Wallabia bicolor* | 1 | | 1 | |
| Tawny frogmouth | *Podargus strigoides* | 1 | 1 | | |
| Wattle bird | *Anthochaera sp.* | 1 | 1 | | |
| Wedge tailed eagle | *Aquila audax* | 1 | | 1 | |
| Common wombat | *Vombatus ursinus* | 2 | | | 2 |

**Table S2.** Detailed outcomes for free-living animal species presented for assessment to field triage stations during the 2019-2020 bushfire season in Victoria, Australia.

| Outcome (after 24 hours at field station) | Total count | Count by species Koala | Other species |
|---|---|---|---|
| Died during care | 4 | 0 | 4 |
| Dead on arrival | 1 | 0 | 1 |
| Euthanised | 52 | 22 | 30 |
| Released | 110 | 101 | 9 |
| Transferred to wildlife carer | 12 | 2 | 10 |
| In owner's care (dogs) | 3 | 0 | 3 |
| Transferred to wildlife hospital* | 3 | 2 | 1 |
| Treated on site | 70 | 65 | 5 |
| Unknown | 4 | 4 | 0 |
| **Total** | **259** | **196** | **63** |

*Wildlife hospital at Zoos Victoria (Healesville Sanctuary or Melbourne Zoo)

**Table S3.** Free-living animals with evidence of direct impact of fire (burns, singes to fur or substantial soot present on assessment) or indirect impact through dehydration only, assessed at field triage stations during the 2019-2020 bushfire season in Victoria, Australia.

| Species | Scientific name | Count of animals presented: Direct fire impacts | Dehydration only | Total animals presented due to likely effects of fire |
|---|---|---|---|---|
| Australian boobook | *Ninox boobook* | 1 | 0 | 1 |
| Common brushtail possum | *Trichosurus vulpecula* | 2 | 0 | 2 |
| Eastern Grey Kangaroo | *Macropus giganteus* | 10 | 0 | 10 |
| Feathertail glider | *Acrobates pygmaeus* | 0 | 3 | 3 |
| Grey headed flying fox | *Pteropus poliocephalus* | 0 | 2 | 2 |
| Southern koala | *Phascolarctos cinereus victor* | 86 | 14 | 100 |
| Lace Monitor | *Varanus varius* | 1 | 0 | 1 |
| New Holland Honeyeater | *Phylidonyris novaehollandiae* | 0 | 1 | 1 |
| Red-bellied black snake | *Pseudechis porphyriacus* | 1 | 0 | 1 |
| Red-necked wallaby | *Macropus rufogriseus* | 0 | 1 | 1 |
| Common ringtail possum | *Pseudocheirus peregrinus* | 1 | 0 | 1 |
| Sugar glider | *Petaurus breviceps breviceps* | 1 | 0 | 1 |
| Superb lyrebird | *Menura novaehollandiae* | 1 | 0 | 1 |
| Wattle bird | *Anthochaera sp.* | 0 | 1 | 1 |
| **Total** | | **104** | **22** | **126** |
